# Genotyping of *Toxoplasma gondii* Isolates from Soil Samples in Tehran, Iran

**Published:** 2013

**Authors:** M Tavalla, H Oormazdi, L Akhlaghi, S Shojaee, E Razmjou, R Hadighi, AR Meamar

**Affiliations:** 1 Dept. of Medical Parasitology, Ahvaz Jundishapur University of Medical Sciences, Ahvaz, Iran; 2 Dept. of Parasitology and Mycology, School of Medicine, Tehran University of Medical Sciences, Tehran, Iran; 3 Dept. of Medical Parasitology and Mycology, School of Public Health, Tehran University of Medical Sciences, Tehran, Iran

**Keywords:** *Toxoplasma gondii*, SAG2, Soil, Genotype, Iran

## Abstract

**Background:**

The protozoan parasite *Toxoplasma gondii* can infect any warm blooded nucleated cells. One of the ways for human infection is ingestion of oocysts directly from soil or via infected fruits or vegetables. To survey the potential role of *T. gondii* oocyst in soil samples, the present study was conducted in Tehran City, Iran.

**Methods:**

A total of 150 soil samples were collected around rubbish dumps, children's play ground, parks and public places. Oocysts recovery was performed by sodium nitrate flotation method on soil samples. For molecular detection, PCR reaction targeting B1 gene was performed and then, the positive results were confirmed using repetitive 529 bp DNA fragment in other PCR reaction. Finally, the positive samples were genotyped at the SAG2 locus.

**Results:**

*Toxoplasma* DNA was found in 13 soil samples. After genotyping and RFLP analysis in SAG2 locus, nine positive samples were revealed type III, one positive sample was type I whereas three samples revealed mixed infection (type, I & III).

**Conclusion:**

The predominant genotype in Tehran soil samples is type III.

## Introduction

*Toxoplasma gondii* is a widely distributed coccidian parasite that can infect a wide range of animals and humans. It is over 100 years since the discovery of the parasite in 1908 and now it is used extensively as a model for cell biology of apicomplexan organisms (1, 2). This coccidian parasite is the causative agent of toxoplasmosis, one of the most prevalent parasitic infectious diseases in animals and humans (3). Transmission of this parasite occurs by consumption of raw or undercooked meat containing tissue cyst or by ingestion of mature oocysts from environmental sources such as soil, water, fruits and vegetables (4). It is estimated that 15% to 85% of human population in the world are chronically infected with *T. gondii* (2). Toxoplasmosis is mainly asymptomatic in immunocompetent individuals, but in immunocompromised patients such as HIV and organ transplantation patients manifestation of clinical signs can be Life threatening (5). Despite of the worldwide distribution of this parasite, there is only one species (*T. gondii*) that causes toxoplasmosis (1). Prevalence of this parasite had been shown to be up to 50% in Iran, which is verified from different parts of the country (6).

*Toxoplasma* oocysts are resistant to environmental conditions and may remain infective for more than one year in different types of soils (4, 7).

Soil contamination with oocysts is related to distribution of infected cat feces in environment. Areas such as gardens, park and around rubbish dump are main places that cats may excrete feces in soil (8).

According to the different methods of characterization such as restriction fragment length polymorphism (RFLP), isoenzyme electrophoresis and random amplified polymorphism, *T. gondii* strains classified into three clonal lineages (genotypes I, II and III) and some atypical genotypes (9–12). It was revealed that three lineages of this parasite have less than 1% difference in genomic level (13). Several genetic markers are available to identify genotypes of *T. gondii* isolates, that the polymorphic surface antigen two (SAG2) is one of the locuses used for differentiation of these three clonal lineages (12, 14).

Genetic analysis of *T. gondii* infection in soil and other environmental resources is of importance to comprehend the epidemiology, patterns of transmission and clonal diversity of the parasite in different parts of the world. One of the studies conducted to environmental contamination with this parasite is the survey of Lass et al. in Poland, that he detected *T. gondii* oocysts in soil samples and confirmed it by molecular methods (15).

The present study was performed to identify *T. gondii* oocysts in soil samples from Tehran, Iran by molecular method and genotyping of positive samples in SAG2 locus by endonuclease enzymes.

## Materials and Methods

### Collection of soil samples

One hundred and fifty soil samples were collected from September 2008 to March 2009 from different parts of Tehran city, such as parks, public places, children's play ground and areas around rubbish dumps. Each sample was weighted about 300 gram which was collected from 3 cm of ground depth. Soil samples were dried at laboratory temperature for 48 hours, sieved and concentrated with modified sodium nitrate flotation as described previously (16).

### Toxoplasma gondii control standard strains

Three strains were obtained from School of Public Health, Tehran University of Medical Sciences. Tachyzoites of *T. gondii* RH strain (type I), tissue cysts of Tehran strain (type II) that was previously isolated from human lymphadenitis (17), and tachyzoites of a virulent strain of *T. gondii* with unknown genotype which is maintain by serial intrapretoneal passages in Department of Parasitology in Tehran University of Medical Science. The strain is introduced as U strain in here.

The tachyzoites were collected from peritoneal cavity of BALB/c mice that were infected three days earlier.

Tissue cysts of Tehran strain (type II) was obtained from brain of BALB/c mice that were injected with bradyzoites of the strain two months earlier.

### DNA Extraction

DNA extraction was performed with the commercial genomic mini kit (A & A Biotechnology, Gdynia, Poland) according to manufacturer's instructions. From each samples 100 µl of DNA was eluted and stored at -20°C until use.

### Detection of Toxoplasma gondii oocyst by PCR

The target of PCR was the 199 bp fragment of the highly conserved 35 fold repetitive B1 gene (AF179871). For PCR reaction, a pair of primer Toxo1 (5’ GGA ACT GCA TCC GTT CAT GAG 3’) and Toxo2 (5’ TCT TTA AAG CGT TCG TGG TC 3’) were used. The PCR was performed according to Schwab et al. (18). To confirm the results, all positive samples were also examined by another pair of primer Toxo-F (5’ AGG CGA GGG TGA GGA TGA 3’) and Toxo-R (5’ TCG TCT CGT CTG GAT CGC AT 3’). These primers are specific for a 200 to 300 fold repetitive fragment of 134 bp (AF 146527) (19, 20). In this study Taq DNA PreMix (Accupower™, BioNeer, South Korea) was used. With the final reaction volume of 25 µl, the amplification was performed by 10 min at 95°C initial step, followed by 30 cycles: denaturation for 5 s at 95° C, annealing for 10 s at 60° and extension for15 s at 72°C. PCR products were analyzed by electrophoresis on 2% agarose gel and stained with ethidium bromide.

### Genotyping and RFLP

For nested-PCR reaction, outer and inner primers for SAG2 locus (3’ and 5’ end) that were previously designed by Howe et al. (12), were used (Table 1).

**Table 1:** Names and sequences of the polymerase chain reaction primer pairs used for Nested-PCR

PCR Reaction		Primer name and sequence
**5’ SAG2 primary PCR**	SAG2 F4	5’-GCTACCTCGAACAGGAACAC-3’
	SAG2 R4	5’-GCATCAACAGTCTTCGTTGC-3’
**3’ SAG2 primary PCR**	SAG2 F3	5’-TCTGTTCTCCGAAGTGACTCC-3’
	SAG2 R3	5’-TCAAAGCGTGCATTATCGC-3’
**5’ SAG2 secondary PCR**	SAG2 F	5’-GAAATGTTTCAGGTTGCTGC-3’
	SAG2 R2	5’-GCAAGAGCGAACTTGAACAC-3’
**3’ SAG2 secondary PCR**	SAG2 F2	5’-ATTCTCATGCCTCCGCTTC-3’
	SAG2 R	5’-AACGTTTCACGAAGGCACAC-3’

**Fig. 1:**
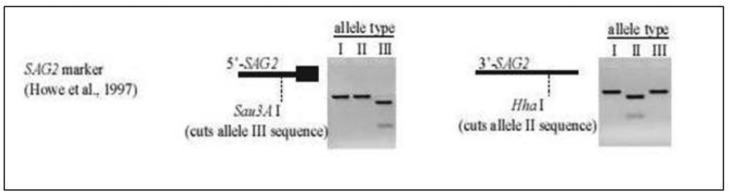
Schematic image for function of restriction enzymes on SAG2 locus (Howe et al. (24)

Length of selected fragments was 241-bp and 221-bp in 5’ end and 3’ end, respectively. The protocol for temperature cycling was used as described by Aspinal et al. (21). In order to distinguish *T. gondii* genotypes, restriction enzymes were used as described previously (12, 22, 23). Two restriction enzymes were selected for RFLP, *Sau3a*I and *Hha*I (Fermentas, Germany). *Sau3a*I enzyme was used for digestion of 5’ end of amplification products that distinguished allele 3 (genotype III) from alleles I and II (genotypes I & II). *Hha*I enzyme was used for digestion of 3’ end of amplification products that distinguished allele II from types I and III (Fig. 1). For RFLP procedure, 10 µl of nested-PCR products of the 5’ and 3’ ends were digested using 3U of *Sau3a*I and *Hha*I restriction enzymes in separate reactions with a total volume of 30 µl at 37° C. Then the fragments were analyzed by 3% agarose gel electrophoresis.

### Sequencing

After nested-PCR, all the positive samples were sequenced by BioNeer Lab (South Korea).

## Results

### Identification of T. gondii oocysts in soil samples

**Fig. 2:**
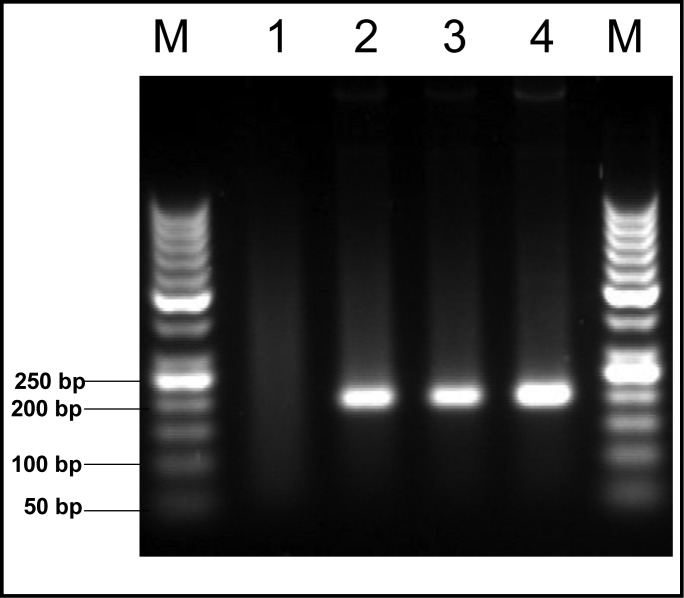
B1 gene amplification products (194 bp) of *T. gondii* on agarose 2%. Lane M, molecular weight marker 50 bp (Fermentas); Lane 1, Negetive control; Lane 4, positive control (RH strain); Lane 2-3 positive soil samples

**Fig. 3:**
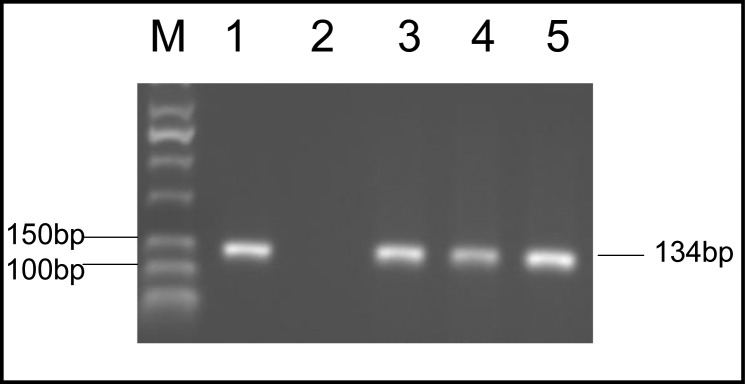
Amplification of 134-bp fragment *T. gondii* from 200 to 300 folds repetitive 529 bp DNA elements on agarose 2%. Lane M, molecular weight marker 50 bp; Lane 1, positive control (RH strain); Lane 2, Negetive control; Lane 3-5, positive soil samples

From 150 soil samples that examined by two pairs of primer in two steps of PCR, 13 samples (8.7%) were positive. In the first step, a 194-bp fragment of B1 gene was amplified (Fig. 2). In the second step, all positive samples were examined by other pairs of primers. The length of fragment in this step was 134-bp from 200 to 300 fold-repetitive elements (AF146527) (Fig. 3). All soil samples were examined twice in each PCR reaction.

### Genotyping of positive samples

All of the 13 positive soil samples were examined by nested-PCR at 5’ and 3’ end of SAG2 locus by four pairs of specific primers.

**Fig. 4:**
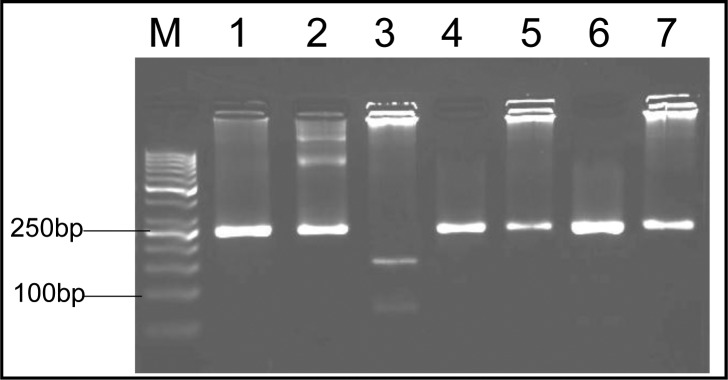
Nested-PCR amplification of SAG2 locus at 3’ end. Lane M, molecular weight marker (50 bp); Lane 1-3, positive controls (RH, unknown and Tehran strains, respectively); Lanes 4-7, soil samples

**Fig. 5:**
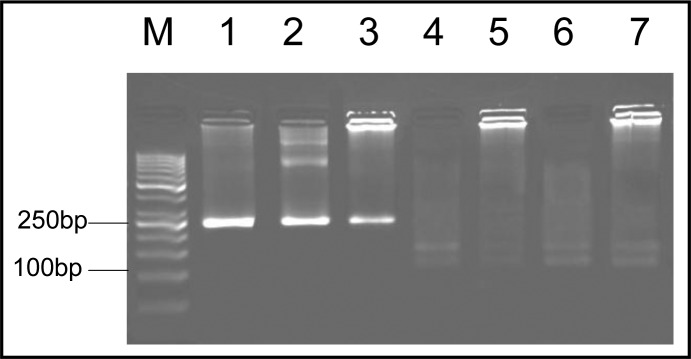
Nested-PCR amplification of SAG2 locus at 5’ end. Lane M, molecular weight marker (50 bp); Lane 1-2, positive controls (RH and Tehran strains, respectively); Lane 3, unknown strain (genotype I); Lanes 4-7, soil samples (genotype III)

**Fig. 6:**
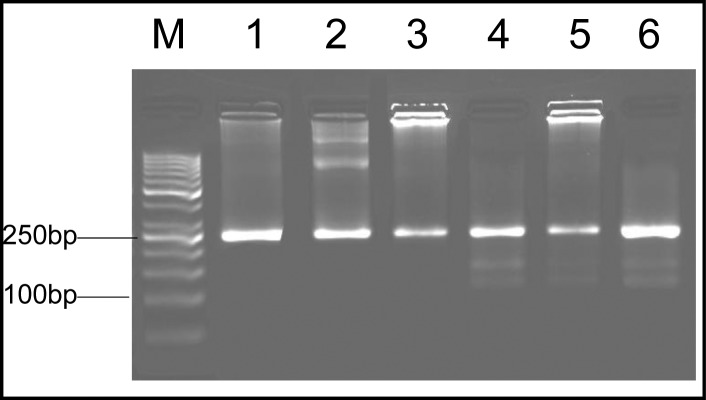
Nested-PCR amplification of SAG2 locus at 5’ end. Lane M, molecular weight marker (50 bp); Lane 1-2, positive controls (RH and Tehran strains, respectively); Lane 3, soil sample (genotype I); Lane 4-6, soil samples (mix genotype of I and III)

These samples showed a 241-bp amplified band in 5’ end of SAG2 locus and a 221-bp amplified band in 3’ end, respectively. After nested-PCR the amplified fragments were used for RFLP. Genotyping of 13 positive soil samples showed that 9 were type III (69%), 3 were mixed of type I and III (23%) and 1 of the samples was type I (8%) (Figs. 4, 5, 6). The pattern of digestion for RH strain and Tehran strain were corresponded to genotypes I and II, respectively. In addition, genotype of U strain was identified type I. All the steps, including nested-PCR and RFLP were performed twice.

### Results of sequencing

The products of the first step of nested-PCR were selected for sequencing and sent to BioNeer Company (South Korea). Sequences were aligned using DNASIS MAX software (Version 3.00; Hitachi, Yokohama, Japan). The nucleotide sequences alignment of samples that had shown type III, revealed the 99-100% homology with other reported sequences in GenBank. The nucleotide sequences data from this paper were submitted in the DDBJ/EMBL/Gene Bank nucleotide sequence databases at accession numbers of AB667972-AB667975.

## Discussion

There is little information about the presence of *T. gondii* oocyst in the soil. In the epidemiological point of view, soil is a large and important source of *T. gondii* infection (4, 24, 25). It is obvious that the abundance of stray cats have a main role in environmental contamination with *T. gondii* oocysts. The number of oocysts shed by naturally infected domestic cats is largely unknown and both young (< 6 mo) and older (>6 mo) cats have been found shedding oocysts in nature (2). *Toxoplasma* oocysts are resistance to environmental factors, and remain in soil up to one year (4). Unfortunately, detection of low amounts of oocysts in soil samples is difficult. According to the report of Lelu et al., factors such as dry or humidity of soil had no significant effect on oocyst recovery (26). On the other hand, *Toxopalsma* oocyst has a resistant wall consisted of several layers, which causes difficult DNA extraction.

*Toxoplasma gondii* strains classified into three genotypes including I, II, III and some atypical genotypes (9–12). It is obvious that the prevalence of three genotypes is variable in different parts of the world. For example in the study of Howe and Sibley, type III was more common in animals than in human toxoplasmosis (27). Dubey et al. showed the predominance of type I in free- ranging chickens from Brazil (28). In another study, the predominant genotype was type III in free- ranging chickens and ducks from Egypt (29). Genotype I predomination was shown in the study of soil samples from three cities in Poland (15). It seems that ecological and geographical conditions could lead to the different prevalence of these types in different regions.

The present study was performed to investigate *T. gondii* genotypes in soil samples from Tehran, Iran for the first time. From 13 positive samples, 9 were type III (69%), 3 were mixed of type I and III (23%) and 1 of the samples was type I (8%). Zia-Ali et al. found the type III as dominant genotype in bird hosts in Iran (30); also he showed the distribution of 70% of type III and 30% of type II in animals (31). Behzadi et al. had reported 85.7% (18/22) of human and mice isolates infected with type II (32). Although the resources of our sampling are different from Zia-Ali et al., the results of these two studies are correlated.

## Conclusion

*Toxoplasma gondii* detection and genotyping with molecular methods such as multilocus PCR-RFLP is possible directly in soil samples. Type III is predominant in Tehran soil samples. More studies regarding to determination of the clonal patterns of *T. gondii* isolates in soil samples by several markers are recommended.
